# On Renal Calculus

**Published:** 1897-04-03

**Authors:** E. Percy Paton

**Affiliations:** Surgical Registrar to Westminster Hospital, &c.


### ON RENAL CALCULUS.

By E. Percy Paton, M.D., M.S., F.R.C.S., Surgical Registrar to Westminster Hospital, &c.

Only of comparatively recent years has the subject of renal calculus been brought very prominently before the attention of surgeons from the success attained in the treatment of this often most painful disease by the operation of nephro-lithotomy.

Previous to the last fifteen or twenty years common stones were only removed from the kidney when they had caused so much irritation as to give rise to suppurative inflammation of the kidney in which they were situated; then followed an abscess in the loose tissue around the kidney, and on opening this the stone which had caused the mischief was accidentally found and removed. Now, however, it is often possible to diagnose and remove a renal stone practically before any destruction of the kidney has taken place.

Stones formed in the kidney are almost always composed either of uric acid or its salts, or of oxalate of lime, except when the urine has become alkaline, when a deposit of phosphates takes place outside a nucleus of either of the above-mentioned materials. The first aggregation of the salts which form a stone usually, in all probability, takes place in the larger tubules of the kidney, but soon from this site of formation finds its way into the pelvis of the organ.

Here it may be found under varied conditions, and upon these conditions will to a large extent depend the symptoms to which it will give rise.

Firstly, the stone may be quite loose in the kidney pelvis, when irritation, pain, &c., will be the symptoms usually set up, or it may be so completely fixed and encysted that no trouble of any sort arises; or again, though fixed, it may still be sufficiently exposed in the pelvis to set up irritation, causing pyelitis, &c., and as it gradually enlarges may practically fill up the kidney pelvis and project as an irregular mass into its various infundibula, so that any urine secreted by the gradually diminishing kidney substance which expands over it, finds its way alongside the stone; or lastly, it may pass into and through the ureter, or remain impacted there.

The results on the kidney itself are usually least in cases in which the calculus is either completely encysted or comparatively small and loose, unless in the latter condition the mass becomes occasionally impacted in the ureter, with subsequent slipping back into the pelvis, when a hydronephrosis results. The same result is also produced when permanent impaction takes place in the ureter, though under these circumstances the cyst developed is usually not of such great size as when occasional relief is afforded to its substance by removal of the back pressure for a time, as absolutely continuous pressure produces very rapidly complete atrophy of urine secreting substance. A large calculus practically filling the pelvis usually very soon sets up so much suppuration and irritation as to cause the kidney in which it lies to be converted into a cyst containing foul purulent urine, which may ere long infect the loose cellular tissue around the organ, with the result that a perinephric abscess is formed.

Reference may now be made to the signs and symptoms produced by a stone. One of the most prominent and important is pain, and this often has a typical character which is very characteristic. This typical pain, called renal colic, comes on acutely in the loin of the affected side, and is in many cases so intense as to amount to actual agony. Spreading from its seat of origin, it radiates into the groin and inner side of the thigh, sometimes as far as the knee, into the testicle in the male, which is commonly retracted and may be swollen, and into the labium in the female; it may also radiate across the abdomen towards the umbilicus. The intensity of the pain is such as often to cause severe vomiting, profuse sweating, and even fainting, and once suffered from or even seen is not easily forgotten, the patient rolling himself about and groaning in the intensity of his distress in a manner to impress the most callous observer. A common urinary symptom during the attack is a very frequent desire to micturate. The pain often subsides quite suddenly, as the stone either slips back into the pelvis or escapes into the bladder. If the stone still remains in the kidney, a dull aching more or less continuous pain is often left there.

Frequently no cause can be assigned for the onset of a painful attack; but sometimes it can be traced to some violent exertion, especially of a jolting character.

Blood in the urine is another very important evidence of the presence of stone. It is usually not great in quantity, and often needs the microscope for its detection in the deposit; as a rule, it is most obvious after an attack of colic, or after exertion or jolting movement; most commonly it is intimately mixed with the urine when passed, though occasionally clots which settle to the bottom are found. A moveable calculus is most prone to cause it. Even in the absence of blood, albumen, as a rule in small quantities, may be found, and this apart from the presence of pus.

Pus, too, is often present, sometimes in considerable quantity, and if this be contained in acid urine, it is a strong indication that it comes from the kidney, and calculus pyelitis is the most common cause of pus from this source. Not infrequently, however, in advanced cases, the urine has decomposed and is alkaline, when naturally the pus will be thought most probably to come from the bladder.

Any deposit, also, must be investigated with care, as in it may be found not only blood corpuscles and pus cells, but also crystals of uric acid or oxalate of lime, which will strongly point in the direction of stone.

Physical examination of the patient not infrequently gives negative results. Of course, if there be the slightest reason to suspect it, the bladder must be carefully sounded to exclude the presence of stone there. The loin must then be carefully palpated by the bimanual method, that is with one hand pressing forward the quadratus lumborum (with the kidney upon it) from behind, while the other palpates the abdomen in front, and strives to find any enlargement or tenderness of the organ. Commonly nothing is discovered, or perhaps only some tenderness; but sometimes, on deep percussion, a certain spot can be found where a sudden blow gives rise to a sharp stabbing pain which is very characteristic when discovered. At other times, however, when a good deal of change has taken place in the renal tissue, and some dilatation of the pelvis is present, a distinct renal tumour can be felt.

We must now turn our attention to the diagnosis of the present of renal calculus from other conditions with which it may be confounded. Typical attacks of renal colic such as described above are not likely to be confused with any other condition; but very often the pain though present is not clearly typical, being much less acute, or spreading to only a very slight extent from the loin into any other part. Such pain as this may be caused by very acid urine with only granular deposit of uric acid, but such cases are usually easily cured by alkalies and diuretics. This sort of pain, often called lumbago, is commonly associated with some stiffness and tenderness of the dorsal muscles, generally on both sides, especially marked when the patient stoops, sits down, or rises up.

Recurrent attacks of appendicitis, or what has been called "appendicular colic," have been said to cause mistakes in diagnosis, but it seems doubtful if a true "colic" of the appendix occurs apart from active inflammation, and pain and trouble here will be associated with rise of temperature and some local physical signs, which will prevent mistakes being made. Traces of blood in the urine associated with some slight pain may be found in granular kidneys, so that in any cases where such a condition is likely to be present the specific gravity and quantity of the urine must be investigated and any other signs of this condition be looked for. New growth and tubercular disease of the kidney are the two other conditions concerning which mistakes are likely to arise. In the former, the bleeding is usually a very prominent symptom, much more so than is usually the case in stone. At the same time, a renal tumour is usually early palpable, and the patient's general condition becomes early affected. With tubercular disease the trouble may be considerable. Though hæmaturia is rarely a prominent feature it is sometimes present, and there is commonly a considerable amount of pus in the urine, which is acid; often a tumour is palpable, but against this there are sometimes signs of tubercle to be found elsewhere; the temperature may rise at night in a suggestive manner; but, most important of all, by centrifugalising the urine the tubercle bacillus can be sometimes stained in the deposit by treating it in the same manner as in staining sputum.

Of course, if there is any reason for such measures the bladder must be carefully explored by sound and catheter. Sometimes, as in a case I saw recently, from which a renal stone was ultimately removed, the diagnosis is much assisted by the patient having at some time passed a small calculus per urethram.

The treatment of renal stone may be discussed under the heads of palliative, operative, and prophylactic, the latter being instituted either after the removal of a stone by operation, or in patients in whom it appears probable a stone may develop.

Palliative treatment is all that can be done during the violence of an attack of colic, and consists in relaxation of all the abdominal muscles, the use of warm applications over the lumbar region and abdomen, but especially the use of hypodermic injections of morphia. Should the severity of the pain continue, it may even be necessary to give the patient chloroform until the violence of the paroxysm is over. Apart from the violent pain, palliative treatment consists in rest and the administration of alkalies with a considerable amount of fluid. These given in certain proportions, appear to have some influence in the direction of solution of the stone, and certainly in some cases the symptoms seem to subside under such treatment, at any rate for a time. One cannot, however, depend upon such a result, as in a stone of any size it is very little likely to do good, and unless the symptoms speedily subside there should be no hesitation in operating for the removal of the calculus, for if left it may only cause suppuration in the kidneys or hydronephrosis or one of the other complications which have been already referred to as a result of calculus, which by practically destroying one kidney, not to speak of other troubles which may result, place the patient in a much worse position than he otherwise would be in should late operation be undertaken. The operation for the removal of the stone is known as nephrolithotomy, and though it cannot be described in detail here, may be outlined as exposing the kidney by an incision in the loin; it is then examined by carefully palpating with the fingers or puncturing with a needle to find the situation of the stone, which is then removed by an incision through the renal tissue. This usually bleeds rather freely at first, but soon stops by pressure. If the stone cannot be felt by the fingers or struck with the needle from outside, the pelvis may be explored by a finger passed in through an incision in the renal tissue, and even the ureter may be explored by a sound passed down its canal. After the operation the kidney wound is left to close and the wound through the muscles and skin sutured, a drain being put in for the first day or two. It is important to note that it is always the renal tissue that is incised in this operation and not the kidney pelvis, as in the latter case it is found almost impossible to close the opening, and a urinary fistula results, which is not the case in a wound of the kidney substance.

